# Automated Weaning from Mechanical Ventilation after Off-Pump Coronary Artery Bypass Grafting

**DOI:** 10.3389/fmed.2017.00031

**Published:** 2017-03-21

**Authors:** Evgenia V. Fot, Natalia N. Izotova, Angelika S. Yudina, Aleksei A. Smetkin, Vsevolod V. Kuzkov, Mikhail Y. Kirov

**Affiliations:** ^1^Department of Anesthesiology and Intensive Care Medicine, Northern State Medical University, Arkhangelsk, Russia

**Keywords:** automated weaning, INTELLiVENT-ASV, coronary artery bypass grafting, monitoring, mechanical ventilation

## Abstract

**Background:**

The discontinuation of mechanical ventilation after coronary surgery may prolong and significantly increase the load on intensive care unit personnel. We hypothesized that automated mode using INTELLiVENT-ASV can decrease duration of postoperative mechanical ventilation, reduce workload on medical staff, and provide safe ventilation after off-pump coronary artery bypass grafting (OPCAB). The primary endpoint of our study was to assess the duration of postoperative mechanical ventilation during different modes of weaning from respiratory support (RS) after OPCAB. The secondary endpoint was to assess safety of the automated weaning mode and the number of manual interventions to the ventilator settings during the weaning process in comparison with the protocolized weaning mode.

**Materials and methods:**

Forty adult patients undergoing elective OPCAB were enrolled into a prospective single-center study. Patients were randomized into two groups: automated weaning (*n* = 20) using INTELLiVENT-ASV mode with quick-wean option; and protocolized weaning (*n* = 20), using conventional synchronized intermittent mandatory ventilation (SIMV) + pressure support (PS) mode. We assessed the duration of postoperative ventilation, incidence and duration of unacceptable RS, and the load on medical staff. We also performed the retrospective analysis of 102 patients (standard weaning) who were weaned from ventilator with SIMV + PS mode based on physician’s experience without prearranged algorithm.

**Results and discussion:**

Realization of the automated weaning protocol required change in respiratory settings in 2 patients vs. 7 (5–9) adjustments per patient in the protocolized weaning group. Both incidence and duration of unacceptable RS were reduced significantly by means of the automated weaning approach. The FiO_2_ during spontaneous breathing trials was significantly lower in the automated weaning group: 30 (30–35) vs. 40 (40–45) % in the protocolized weaning group (*p* < 0.01). The average time until tracheal extubation did not differ in the automated weaning and the protocolized weaning groups: 193 (115–309) and 197 (158–253) min, respectively, but increased to 290 (210–411) min in the standard weaning group.

**Conclusion:**

The automated weaning system after off-pump coronary surgery might provide postoperative ventilation in a more protective way, reduces the workload on medical staff, and does not prolong the duration of weaning from ventilator. The use of automated or protocolized weaning can reduce the duration of postoperative mechanical ventilation in comparison with non-protocolized weaning based on the physician’s decision.

## Introduction

The discontinuation from mechanical ventilation after coronary artery bypass grafting (CABG) may prolong and significantly increase the load on intensive care unit (ICU) personnel. To date, we possess several options for optimization of weaning and reducing duration of mechanical ventilation. One of these approaches includes the use of weaning protocols. Protocolized weaning is able to decrease duration of postoperative mechanical ventilation and ICU stay as compared to non-protocolized discontinuation of ventilation in different categories of patients (1, 2). However, there are some difficulties with its preparation, implementation it in routine clinical practice, and compliance by the hospital staff (3). To overcome these problems, the computerized weaning protocols aiming to facilitate fast track and optimize discontinuation of ventilation have been introduced into the clinical practice (4–6).

The alternative to the computerized weaning protocol can be different automated modes (5, 7, 8). One from these modes, INTELLiVENT-ASV is an automated algorithm based on the principle of the least work of breathing. Ventilation settings including minute volume, tidal volume (V_T_), and respiratory rate (RR) are adjusted automatically to reach a target end-tidal CO_2_ (EtCO_2_) in passive patients and a target RR in active patients. Oxygenation settings [inspiratory fraction of oxygen (FiO_2_) and positive end-expiratory pressure (PEEP)] are adjusted automatically to reach a target oxygen saturation measured by pulse oximetry (SpO_2_). The INTELLiVENT-ASV mode provides automatic correction of respiratory parameters for the achievement of the optimal respiratory pattern and includes an additional function of the automated weaning protocol (Quick Wean) (7). The Quick Wean algorithm progressively decreases pressure support (PS), screens for the readiness-to-wean criteria, and automatically conducts fully controlled spontaneous breathing trials (SBTs). INTELLiVENT-ASV was introduced in 2010; its efficacy and safety have been evaluated in several studies, including on-pump cardiac surgery and critically ill patients (7, 9, 10).

The patients with CABG represent the majority of adult cardiosurgical population (10). Patients after off-pump coronary artery bypass grafting (OPCAB) are usually easier to wean, and they have less postoperative respiratory complications in comparison with on-pump surgery (11, 12). However, the number of patients operated with OPCAB technique reaches 60–100% in some regions (13) leading to increased load on the personnel of cardiosurgical ICU. The role of INTELLiVENT-ASV mode during weaning from ventilation after OPCAB is still unsettled. Thus, it is important to assess efficacy and safety of the automated weaning mode in the off-pump coronary patients (12).

We hypothesized that automated mode using INTELLiVENT-ASV can decrease duration of postoperative mechanical ventilation, reduce workload on medical staff, and provide safe ventilation after off-pump coronary surgery. The primary endpoint of our study was to assess the duration of postoperative mechanical ventilation during different modes of weaning from respiratory support (RS) after OPCAB. The secondary endpoint was to assess safety of the automated weaning mode and the number of manual interventions to the ventilator settings during the weaning process in comparison with the protocolized weaning mode.

## Materials and Methods

The study was performed in a 900-bed university hospital (City Hospital #1 of Arkhangelsk, Russia). During year 2015, 40 adult patients were enrolled into a randomized controlled study. The study design and the informed consent form were approved by the Ethical Committee of Northern State Medical University (Arkhangelsk, Russian Federation) and registered with http://ClinicalTrials.gov (NCT02524522). Written informed consent was obtained from every patient before surgery. Inclusion criteria were the following: elective OPCAB; age between 18 and 80 years; ability to give the informed consent. Exclusion criteria were: morbid obesity with body mass index >40 kg/m^2^, constant atrial fibrillation, hemodynamic instability after admission to the ICU, and complications during surgery.

All patients were intubated using the standard induction technique. Anesthesia was provided using sevoflurane (0.5–3.0 vol. %) to maintain BIS values between 40 and 60 (LifeScope, Nihon Kohden, Japan).

After pre-oxygenation with 80% O_2_ and tracheal intubation, patients were ventilated using a protective volume-controlled mode (Dräger Primus, Germany) with V_T_ of 8 mL/kg of predicted body weight (PBW), flow of 1 L/min, and PEEP of 5 cm H_2_O. The value of FiO_2_ was set to at least 50% or higher to achieve intraoperative SpO_2_ above 95%. The RR was adjusted to maintain an EtCO_2_ value within 30–35 mmHg.

After surgery, all patients were transferred to the postoperative cardiac ICU and shortly sedated with continuous infusion of propofol (2–4 mg/kg/h) to maintain BIS values within 60–70. RS in ICU was provided by a G5 ventilator (Hamilton Medical, Switzerland) using pressure controlled ventilation mode with parameters of intraoperative ventilation.

After the initial measurements, sedation was stopped, and the weaning from RS was initiated. All the patients were randomized into two groups: automated weaning (*n* = 20) and protocolized weaning (*n* = 20). The randomization procedure was performed by means of www.randomizer.org. Patients from the automated weaning group received mechanical ventilation in the INTELLiVENT-ASV mode with automatically adjustment of all parameters, target EtCO_2_ within 30–35 mmHg, and SpO_2_ >95%. We also activated the quick wean option with automatic conduction of SBT. The test lasted 30 min and was automatically interrupted in case of SpO_2_ <90%, RR >30/min, EtCO_2_ >45 mmHg and V_T_ <5.0 mL/kg of PBW. In case of premature interruption of the test, another SBT could be automatically performed 30 min later. In case of prolonged (more than 5 min) desynchronization or RR >35/min, we provided additional analgesia and assessed the necessity for the treatment of metabolic acidosis or hyperthermia. If these methods were ineffective, we changed INTELLiVENT-ASV mode to synchronized intermittent mandatory ventilation (SIMV) + PS mode with manual adjustment of all the settings.

Patients from the protocolized weaning group received mechanical ventilation in the SIMV mode with inspiratory pressure aiming to achieve V_T_ 8 mL/kg of PBW, PEEP of 5 cm H_2_O, and start FiO_2_ 50% or higher to achieve SpO_2_ >95%. If FiO_2_ was >50%, the stepwise attempts to decrease it by 10% were performed every 30 min. The RR was adjusted to maintain an EtCO_2_ value within 30–35 mmHg. The weaning protocol included gradual reduction of inspiratory pressure and mandatory RR. Thus, the ventilator parameters were assessed every 30 min and adjusted, if necessary, aiming at a stepwise decreasing inspiratory pressure/PS by 2–4 cm H_2_O and mandatory RR by 2–4/min. After decrease of mandatory RR to 6/min, inspiratory pressure and PS to 6–8 and 8–10 cm H_2_O, respectively, and FiO_2_ to 50%, the SBT was started. The SBT was performed in the PS mode with PS 6–8 cm H_2_O, PEEP 5 cm H_2_O, FiO_2_ 40%. The duration of the test was 30 min. The SBT was considered to be successful if the patient displayed no episodes of tachypnea (RR >30/min), had V_T_ >6 mL/kg of PBW, SpO_2_ >90%, and had no signs of hemodynamic instability during the last 30 min. In case of SBT failure, another test could be performed only 30 min later. To assess patient safety, we recorded the number and duration of the episodes of the unacceptable ventilation lasting more than 30 s during the entire weaning process (10). The characteristics of safety ventilation zone are described in Table 1. The research assistant was at each patient’s bedside during the entire weaning process to record the time that the patient spend in the unacceptable ventilation zone and to record the number of interventions required to set the ventilator. After successful SBT, all the patients were immediately extubated.

**Table 1:** **Safety ventilation zone (10)**.

Characteristic	Safety ventilation
V_T_, mL/kg PBW	6–10
Ppeak, cm H_2_O	<35
EtCO_2_, mmHg	25–45
RR/min	10–30
SpO_2_ %	>90

*V_T_, tidal volume; PBW, predicted body weight; Ppeak, peak inspiratory pressure; EtCO_2_, end-tidal CO_2_; RR, respiratory rate; SpO_2_, oxygen saturation*.

After the extubation, the patients received a supplementary oxygen flow of 4 L/min *via* a nasal catheter. During the weaning process and in the early post-extubation period, all the patients received multimodal analgesia with continuous infusion of fentanyl and discrete administration of paracetamol, aiming to maintain visual analog scale below 30 mm in rest.

The measurements included ventilator parameters, blood gases (ABL800Flex, Radiometer, Denmark), EtCO_2_, SpO_2_, RR, and pulse rate (Capnostream-20, Covidien, USA). Continuous hemodynamic measurements included ECG monitoring and invasive arterial pressure (Nihon Kohden, Japan). All these parameters were registered after transfer to the ICU, as well as after SBT and at 2, 6, 12, and 18 h after extubation. In addition, we recorded the preoperative EuroScore II, perioperative fluid balance, left ventricle ejection fraction before surgery, duration of postoperative mechanical ventilation, and ICU stay, as well as hospitalization time and chest X-ray the morning after the surgery. The changes in the chest X-rays were diagnosed for any signs of pleural effusion or atelectases. We also assessed the load on medical staff by calculating the number of manual interventions to the ventilator settings during the weaning process.

**Figure 1:**
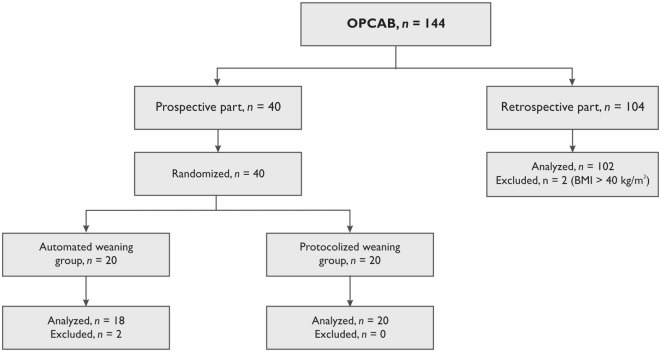
**Study flow chart**. OPCAB, off-pump coronary artery bypass grafting; BMI, body mass index.

We also conducted a retrospective analysis of the ICU charts of 102 patients after OPCAB conducted in 2015. The study flow chart is presented in Figure 1. Patients had all inclusion criteria and no exclusion criteria used in the prospective part of this study. Weaning process in these patients was based on the physician’s decision made without any protocol.

### Statistical Analysis

For data collection and analysis, we used SPSS software (version 17.0; SPSS Inc., USA) and MedCalc software (version 12.3, MedCalc Software, Belgium).

Calculation of sample size was based on our initial observations. To reveal the reduction in duration of postoperative RS by 40% in the automated weaning group compared to the non-protocolized weaning group with α of 0.05, power of 0.8, and the ratio of patients in groups 1:3, the number of patients should be not less than 16 in the automated weaning group and not less than 80 in the non-protocolized weaning group. To detect the decrement of the number of deviations from safety ventilation zone by 50% in the group with automated mode of ventilation compared to the protocolized weaning with α of 0.05, power of 0.8, and the ratio of patients in groups 1:1, the number of patients should be not less than 12 in each group.

All the variables were expressed as median (25th–75th interquartile interval). The groups were compared using Mann–Whitney test. The intragroup comparisons were performed by Friedman and *post hoc* Wilcoxon tests with Bonferroni correction. Nominal data were compared using χ^2^ test and expressed as patient number.

## Results

The main demographic and perioperative characteristics of the studied patients are shown in Table 2. No major bleeding events or the need for resternotomy were observed during the study. Two patients from the automated weaning group were excluded from the analysis because of the protocol deviations. We registered no serious protocol deviations in the protocolized weaning group.

**Table 2:** **Characteristics of the patients**.

Characteristics	Automated weaning (*n* = 18)	Protocolized weaning (*n* = 20)	Standard weaning (*n* = 102)
Age, years	63 (54–70)	60 (55–71)	62 (57–67)
BMI, kg/m^2^	30 (28–33)	29 (27–31)	29 (26–32)
EuroScore II, points	1.24 (0.75–1.56)	1.15 (0.81–1.60)	1.28 (0.76–1.93)
NYHA, points	2 (2–2)	2 (2–2)	2 (2–2)
EF before surgery, %	61 (53–65)	57 (47–63)	56 (51–64)
COPD, number of patients	1	1	3
Dose of the fentanyl during surgery, mg	1.10 (0.85–1.10)	1.10 (1.00–1.30)	1.10 (1.00–1.20)
Duration of surgery, min	218 (178–260)	205 (155–160)	205 (170–235)
PaO_2_/FiO_2_ after ICU admission, mmHg	270 (169–325)	270 (197–347)	227 (178–295)
PaCO_2_ after ICU admission, mmHg	38.8 (35.5–40.8)	39.3 (36.4–41.6)	40.0 (35.0–45.0)
Duration of ICU stay, days	2 (1–3)	1 (1–3)	1 (1–2)
Hospitalization time, days	10 (9–12)	11 (10–14)	11 (10–13)
Changes on chest X-ray, %	17	16	51

*Data presented as median (25th–75th percentile), numbers or percentage*.

*BMI, body mass index; NYHA, New York Heart Association; EF, ejection fraction; COPD, chronic obstructive pulmonary disease; ICU, intensive care unit*.

**Figure 2:**
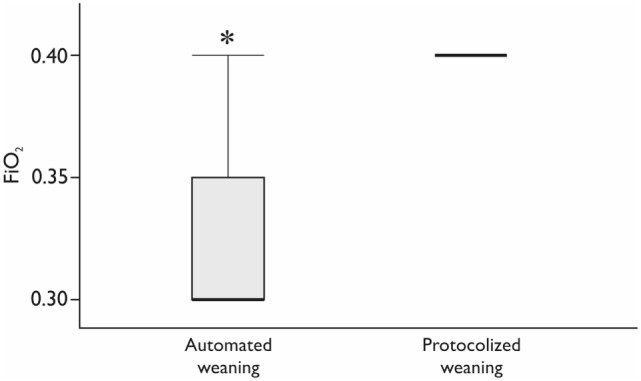
**The fraction of inspired oxygen (FiO_2_) during spontaneous breathing trial**. *Mann–Whitney test, *p* < 0.05. Variables are expressed as median (25th–75th interquartile interval).

The studied groups were comparable at ICU admission and postoperatively (Table 2). All the patients had stable hemodynamics and gas exchange during and after SBT without clinically significant differences (Table 3). The number of SBT did not differ between the groups: 1 (1–2) tests in each group (*p* = 0.39). Fraction of inspired oxygen during SBT was lower by 10% in the automated weaning group (*p* < 0.01; Figure 2). At the same stage, PaO_2_ was 105 (89.8–112.8) mmHg in the INTELLiVENT-ASV group and 138 (112.5–149.0) mmHg in the SIMV group (*p* = 0.02). All the patients were successfully extubated, none from them required reintubation.

**Table 3:** **Respiratory parameters after spontaneous breathing trial and tracheal extubation**.

Parameter	Group	SBT	2 h	6 h	12 h	18 h
SpO_2_, %	INTELLiVENT-ASV	99 (97–99)	94 (92–97)	94 (91–97)	95 (91–96)	94 (93–96)
	SIMV	99 (97–100)	95 (93–97)	94 (93–97)	96 (93–98)	94 (92–95)
RR/min	INTELLiVENT-ASV	16 (13–19)	15 (15–16)	15 (14–17)	15 (13–19)	15 (14–16)*
	SIMV	16 (15–18)	15 (14–20)	15 (14–20)	15 (13–22)	17 (14–24)
PaO_2_/FiO_2_, mmHg	INTELLiVENT-ASV	324 (246–356)	318 (305–354)	333 (289–355)	314 (276–350)	320 (288–349)
	SIMV	338 (281–353)	346 (309–385)	319 (290–378)	324 (290–362)	292 (266–341)
PaCO_2_, mmHg	INTELLiVENT-ASV	38 (37–42)	39 (35–39)	37 (35–39)	37 (35–39)	37 (32–38)
	SIMV	36 (32–40)	37 (34–38)	37 (33–39)	35 (32–39)	37 (32–38)
EtCO_2_, mmHg	INTELLiVENT-ASV	38 (35–40)	37 (33–39)	36 (35–38)	35 (32–38)	35 (32–38)
	SIMV	37 (32–38)	35 (33–39)	35 (34–37)	34 (32–37)	33 (31–36)

*Data presented as median (25th–75th percentile)*.

*HR, heart rate, MAP, mean arterial pressure, RR, respiratory rate, SBT, spontaneous breathing trial*.

*INTELLiVENT-ASV, automated weaning group; synchronized intermittent mandatory ventilation (SIMV), protocolized weaning group. Data are assessed after spontaneous breathing trial and 2, 6, 12, and 18 h after tracheal extubation*.

*Mann–Whitney test, p < 0.05.

**Figure 3:**
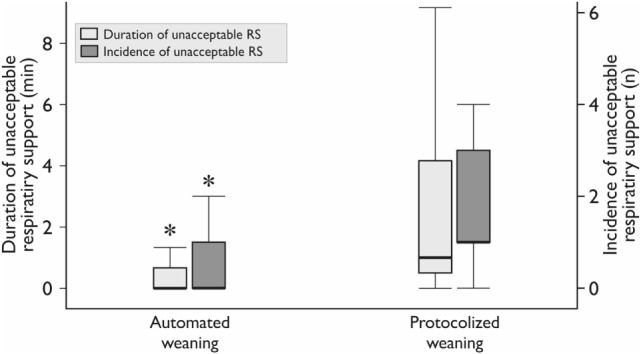
**The duration and incidence of unacceptable respiratory support (RS)**. The incidence is the median number of episodes per patient. *Mann–Whitney test, *p* < 0.05. Variables are expressed as median (25th–75th interquartile interval).

**Figure 4:**
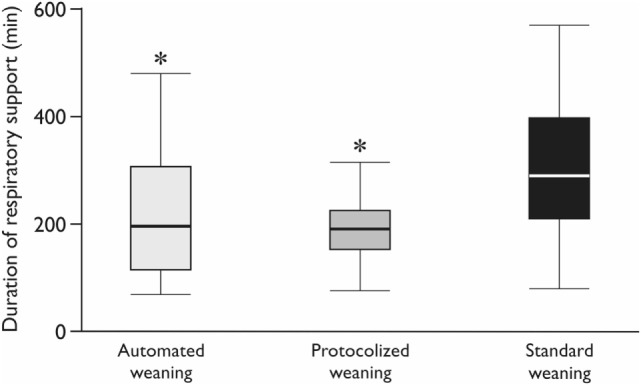
**The duration of postoperative mechanical ventilation**. CMV—controlled mechanical ventilation; *Mann–Whitney test with Bonferroni correction, *p* < 0.017. Variables are expressed as median (25th–75th interquartile interval).

Realization of the automated weaning protocol in 18 patients did not require any changes in respiratory settings vs. 7 (5–9) adjustments per patient in the protocolized weaning group (*p* < 0.001). The number of deviations from the safety ventilation zone and the duration of these episodes were significantly lower in the automated weaning group (*p* = 0.02 and *p* = 0.03, respectively; Figure 3; Table 4). The following deviations were presented: reduced V_T_, 35%; increased RR, 28%; increased EtCO_2_, 15%; increased V_T_, 12%; decreased EtCO_2_, 5%; and decreased SpO_2_, 5%. The duration of postoperative ventilation was similar in the automated weaning group and the protocolized weaning group and was significantly shorter than in the standard weaning group: 193 (115–309) min vs. 197 (158–253) min vs. 290 (210–411) min, respectively (*p* = 0.004 compared with the automated weaning group and *p* = 0.002 compared with the protocolized weaning group, respectively) (Figure 4).

**Table 4:** **The incidence and types of deviations from safety ventilation zone during weaning from mechanical ventilation**.

Characteristics	Automated weaning (*n* = 18)	Protocolized weaning (*n* = 20)	*p*
Total number of deviations	18	41	
Number of deviations per patient	0 (0–1)	1 (1–3)	0.022
Low V_T_, mL/kg	3	11	0.07
High V_T_, mL/kg	1	5	0.58
Low EtCO_2_, mmHg	5	7	0.97
High EtCO_2_, mmHg	6	9	0.75
High RR	3	7	0.48
Low SpO_2_, %	0	2	0.8

*Data presented as median (25th–75th percentile) or numbers*.

*V_T_, tidal volume; EtCO_2_, end-tidal CO_2_; RR, respiratory rate; SpO_2_, oxygen saturation*.

We also observed a tendency for reduction of the number of changes during postoperative chest X-ray, both in the automated weaning group (*p* = 0.12) and in the protocolized weaning group (*p* = 0.098) as compared to the standard weaning group (Table 2). The duration of ICU stay did not differ between the groups (Table 2).

## Discussion

We found that INTELLiVENT-ASV mode was safe in patients after OPCAB, required fewer mechanical ventilator interventions compared to the protocolized weaning strategy, and did not prolong the weaning process. Our study has shown that the automated weaning after OPCAB using INTELLiVENT-ASV was well tolerated by the overwhelming majority of patients. This is consistent with findings of other authors (7, 9, 10, 14, 15). The possible problems during automated weaning can be related with poor quality of SpO_2_ signal during tissue hypoperfusion in patients with vasoconstriction, shock, and cold extremities that requires temporary deactivation of the oxygenation controller (15). The patients with respiratory failure and increased gradient between arterial and end-tidal CO_2_ can require deactivation of the EtCO_2_ controller for 5% of the total ventilation time (15). However, in elective OPCAB, we did not register such problems.

Providing protective mechanical ventilation and avoiding baro-, volumo-, and atelectotrauma is one of the important goals of INTELLiVENT-ASV mode. In our study, automated mode reduced the number and duration of deviations from safety ventilation zone. Similar results were shown in patients with ARDS and in the postoperative period after cardiac surgery (10, 15). In a recent study, the authors showed that INTELLiVENT-ASV required less manual interventions, while delivering safety ventilation in terms of V_T_, SpO_2_, and EtCO_2_ in ICU patients with an expected duration of mechanical ventilation of more than 48 h (16). Lellouche et al. also demonstrated that this fully automated system can be safely used in stable patients following on-pump cardiac surgery, reducing the number of manual interventions and providing protective ventilation (10). In the study of these authors, 43% of conventionally ventilated patients had at least one episode of unacceptable ventilation compared with 13% of patients receiving automated ventilation. The time of unacceptable ventilation was also significantly longer during conventional ventilation (10).

Another goal of protective ventilation is the prevention of hyperoxia. The negative impact of hyperoxia has been studied for many years, and now it is known that excessive oxygen supply may lead to the absorption atelectases and lung damage (17–19). In addition, hyperoxia has been associated with poor outcomes in ICU patients, particularly after cardiac arrest and stroke (18). On the other hand, hyperoxia has several potential benefits including reduction of surgical site infection (20). To date, there are a number of studies supporting rather conservative oxygenation targets in patients receiving mechanical ventilation (19, 21). It was shown that restrictive oxygen supplementation might help to achieve weaning criteria with subsequent extubation faster than liberal oxygenation strategy with increased FiO_2_. However, a recent retrospective database study found that hyperoxia is commonly seen in the ICU and in most cases does not lead to the adjustment of ventilator settings if FiO_2_ is below or equal to 40% (15). We also had FiO_2_ level 40% during SBT in the protocolized weaning group ventilated with SIMV + PS but the patients from the automated weaning group received FiO_2_ 30% avoiding hyperoxia without any negative effects on their respiratory status and SpO_2_ 99% in both groups. This is consistent with the study of Abutbul et al. who demonstrated decreased FiO_2_ in the INTELLiVENT-ASV mode without a significantly reduced PaO_2_ compared with other modes, where FiO_2_ was adjusted manually (22). Providing protective ventilation in automated modes can be explained by the automated continuous monitoring of the patients respiratory status with more frequent automated adjustments of ventilator settings in comparison with traditional modes (13).

Nowadays, protective ventilation is used not only in ARDS patients (23). Several studies show that the use of protective ventilation in perioperative period can decrease the number of complications after surgery in high-risk patients (24, 25). In our study, we also observed the tendency for a decrement of the number of complications, detected by the chest X-rays after OPCAB both in the automated and protocolized weaning groups.

One of the important tasks for automated modes is to decrease the load on medical staff due to automatic adjustments of the ventilator setting. In our study, the number of interactions between physician and ventilator with adjustment of parameters in the protocolized weaning group was much higher than in automated weaning group. According to our protocol, we had to assess respiratory settings every 30 min that significantly increased the load on medical staff and did not decrease the time of weaning compared with the automated mode. These results accompany to the findings of a recent study of patients after cardiac surgery, which has also shown that INTELLiVENT-ASV required less number of interactions than conventional mode (14).

One of the end-points of the study was the comparison of the postoperative ventilation time. Previous studies are not providing equivocal point of view regarding the influence of the automated weaning modes on the duration of postoperative mechanical ventilation (9). According to the recent meta-analysis, the use of the automated modes can decrease the duration of mechanical ventilation in therapeutic, but not surgical ICU (26). The problem with interpretation of these results can be associated with different comparison groups, different modes of ventilation and different study protocols. Thus, the use of the automated mode (SmartCare™) did not lead to the reduction of the weaning process and the period of mechanical ventilation, as well as the length of ICU stay and hospitalization time in comparison with the physician-directed protocolized weaning (5). In our study, the duration of postoperative ventilation also did not differ between automated weaning and protocolized weaning groups. However, the absence of any weaning protocol in the retrospectively evaluated group of OPCAB patients has prolonged the discontinuation of ventilation by 100 min in comparison with the physician-directed protocol and automated weaning. Only few studies in medical patients were able to show the influence of the automated modes on the length of ICU stay (5). No benefits were demonstrated in surgical ICU (26) that also corresponds to the results of our study.

Our study has several limitations related to the differences in study algorithms and confounding factors affecting weaning from ventilator. Moreover, this single-center study has a limited number of observations, used retrospective analysis for the non-protocolized weaning, and was not powered for demonstrating the reduction in number of complications and in duration of ICU and hospital stay in the automated weaning group.

## Conclusion

The automated weaning system after off-pump coronary surgery might provide postoperative ventilation in a more protective way, reduces the workload on medical staff, and does not prolong the duration of weaning from ventilator. The use of automated or protocolized weaning can reduce the duration of postoperative mechanical ventilation in comparison with non-protocolized weaning based on the physician’s decision.

## Author Contributions

All authors have contributed equally to this work.

## Conflict of Interest Statement

The authors declare that the research was conducted in the absence of any commercial or financial relationships that could be construed as a potential conflict of interest. The reviewer MS and handling Editor declared their shared affiliation, and the handling Editor states that the process nevertheless met the standards of a fair and objective review.
